# The Radical Cure of Bunions

**Published:** 1902-05-17

**Authors:** A. H. Tubby

**Affiliations:** Surgeon to the Westminster and National Orthopædic Hospitals.


### Hospital Clinics and Medical Progress.

### THE RADICAL CURE OF BUNIONS.

By A. H. Tubby, M.S., Surgeon to the Westminster and National Orthopædic Hospitals.

The effectual treatment of this most painful deformity of the foot has long been a matter of great difficulty, largely owing to the fact of not only the soft parts being involved but also the bony structures. In fact the term "bunion," although usually limited to the swelling formed by the subcutaneous tissues and false bursa, should be extended to include the hypertrophied head of the first metatarsal bone and the overgrown base of the first phalanx.

The commencement of a bunion is in a slight dislocation outwards of the base of the first phalanx, leaving the inner part of the head of the first metatarsal bone bare, and on the latter prominence a bursa forms. As the dislocation proceeds the extensor proprius pollicis tendon is displaced outwards until it forms the base of a triangle, the two remaining sides of which are composed of the first phalanx and the first metatarsal bone. When the displacement is well marked, hypertrophy of the inner side of the first metatarsal bone takes place, and oftentimes a distinct exostosis appears. Whether this be due to irritation, or—as I strongly suspect—to changes of an osteo-arthritic nature, I am somewhat uncertain, but there is no doubt that much aggravation of pain ensues when the exostosis is at all large. Owing to the continued pressure of the boots, corns often form on the skin, and when inflammation of the false bursa ensues, intolerable pain results. It is still a moot point why the affection is so much more common in women than in men, and why it begins at a much earlier date in the former. I have seen it commencing as early as the fourteenth and fifteenth years in girls.

In slight cases relief may be obtained by various devices, it being premised that in all instances sensible boots are worn. A small pad of cotton wool placed between the first and second toes will relieve early cases. In others a little more severe a bunion spring may be worn, and the pain of the bunion can be subdued by the application either of belladonna and glycerine or by painting it with tincture of iodine. But too often, in spite of all palliative treatment, the enlargement of the head of the metatarsal bone continues, and some further measure must be carried out. Bearing in mind that the underlying factor in all these cases is enlargement of the head of that bone, often of an osteophytic nature, the only plan likely to afford permanent relief is to attack the bone itself. Some surgeons have tried the effect of chiselling off the internal prominence, but this is ineffectual because the enlargement also affects the other aspects of the head of the metatarsal bone; while excision of the false bursa is of course useless, since it only forms again. Other surgeons have tried removal of a wedge-shaped piece from the neck of the first metatarsal bone, but this method fails.

There is, I believe, only one method of radical cure in these cases, and that is excision of the head of the first metatarsal bone. It is rarely necessary to touch the base of the phalanx, and, indeed, it is better to avoid it because ankylosis of the joint is likely to ensue. The method which I have adopted in over 60 cases, all of which have been successful, is as follows:—An incision of about two to three inches long is made on the inner side of the great toe with its centre opposite the most prominent part of the head of the metatarsal bone. The incision may be straight or curved, or be made so as to include the piece of skin covering the false bursa. The incision is then deepened down to the bone and the false bursa removed. If it be inflamed great care must be taken not to allow its contents to escape and be distributed in the deeper tissues. The soft parts on either side of the incision are then reflected well outwards until the outer side of the bone is reached, due regard being had to the integrity of the extensor proprius pollicis tendon. This it is not necessary to divide. The joint is then freely opened, the ligaments being completely divided. With a small saw—Adams' being the best—the neck of the first metatarsal bone is divided just behind the articular cartilage. The direction of the division of the bone is of importance. It should be oblique from above downwards and before backwards. The head of the bone can now be removed with a few touches of the knife. The sharp edges of the neck of the first metatarsal bone are then trimmed off with a small pair of bone forceps especially underneath and on the outer side. Underneath so that the edge shall not worry the sesamoid bones, and on the outer side so that there shall be no sharp pressure on the branches of the internal plantar nerve. All hæmorrhage having been arrested the wound is closed. A pad is placed between the first and second toes so as to ensure that when healing takes place the first toe shall be in a right line. In most cases it is better to place beneath the great toe a small malleable iron splint curved in such a way that the great toe shall point slightly upwards. After a week to ten days the stitches are removed and passive movements begun, and generally in two to three weeks the patient is able to get about.

The final results of this operation are fully justified. The foot is of almost normal appearance, the only point noticeable about it being that the great toe is slightly shorter than the second, but it is in a straight line and has free movement; in fact often better than it had previous to the operation. In only three cases have I known pain to occur afterwards, but this I believed to be due to adhesions; for with movement under gas and subsequent massage all pain disappeared. In fact so sure do I consider the results of this operation to be, and so efficient is it as a curative measure, that I have on two occasions removed the heads of both first metatarsal bones for bunions in dancing masters who were dependent upon the usefulness of their feet for their livelihood. I would therefore strongly recommend this operation as one without risk, and as being certain to cure a very troublesome and disabling deformity.

